# Outcomes of Patients With Appendiceal Neoplasms Based on Clinicopathological Features and Surgical Intervention: A Retrospective Observational Study at a Tertiary Cancer Centre in India

**DOI:** 10.7759/cureus.114520

**Published:** 2026-08-14

**Authors:** Nikhil Garg, Senthilkumar Moorthy, Vikas Warikoo, Varun M, Ajinkya Pawar, Rajan Yadav

**Affiliations:** 1 Surgical Oncology, All India Institute of Medical Sciences, Bhatinda, Bhatinda, IND; 2 Surgical Oncology, The Gujarat Cancer and Research Institute, Ahmedabad, IND; 3 Gastrointestinal and Hepatopancreaticobiliary Oncosurgery, The Gujarat Cancer and Research Institute, Ahmedabad, IND; 4 Surgical Oncology, Aster Medicity, Bangalore, IND

**Keywords:** appendicectomy, cytoreductive surgery, hipec, histology subtypes, lamn, overall survival, pseudomyxoma peritonei, tumor stage

## Abstract

Introduction

Tumour biology remains the key determinant of prognosis in appendiceal neoplasms. In this study, we analyse the clinicopathological characteristics and outcomes of patients with appendiceal neoplasms treated at our institution, with particular emphasis on overall survival among different tumour types.

Material and methods

Patients with histologically confirmed appendiceal neoplasms treated between January 2021 and December 2025 were included. Clinical, pathological, treatment, and follow-up data were retrieved from medical records and institutional databases. Variables analysed included age, gender, histological subtype, disease stage, treatment modality, recurrence status, and survival outcomes. The primary endpoint was overall survival (OS) among treated appendiceal neoplasms with different tumor subtypes. A subgroup analysis was also performed to study the difference in treatment outcomes in patients with low-grade appendiceal mucinous neoplasm (LAMN) and their overall survival.

Results

Among the 55 patients in our study, the following observations were made. The median age of patients at presentation was 54 years [95% confidence interval (CI): 46-61], with a slight female predominance, 30(54.5%). The majority of patients, 37 (67.3%), had LAMN, followed by eight (14.5%) with high-grade appendiceal mucinous neoplasm (HAMN), eight (14.5%) with adenocarcinoma, and two (3.6%) with signet ring adenocarcinoma. Many patients presented with advanced disease, with stage IV accounting for 53.7% of cases (22 out of 41 patients had stage 4 disease). Overall survival differed significantly by histopathology. Patients with LAMN histology had better survival compared with other histologic subtypes (log-rank p = 0.026). In Cox proportional hazards analysis, LAMN was also associated with a lower risk of death [hazard ratio (HR) = 0.24, p = 0.040]. Among patients with LAMN histology, overall survival did not differ significantly between those undergoing appendectomy alone and those undergoing other surgical procedures (log-rank p = 0.48). Both groups demonstrated high survival rates (> 85%) up to three years, with very few deaths observed.

Conclusion

Our findings demonstrate that survival in treated appendiceal neoplasms is predominantly influenced by tumour biology, particularly histological risk group and metastatic status at presentation. Patients with low-grade mucinous neoplasms experienced relatively favourable outcomes, often surviving without recurrence, whereas high-risk histology variants were associated with poorer survival and increased incidence of peritoneal dissemination.

## Introduction

Tumour biology remains the key determinant of prognosis in appendiceal neoplasms [1]. Recent large database studies have demonstrated an increasing incidence of appendiceal neoplasms, likely attributable to improved imaging, heightened clinical awareness, and more frequent pathological examination of appendectomy specimens [2,3]. Management protocols for these tumours have also evolved substantially in recent years. The current classification of appendiceal neoplasms recognises distinct pathological entities with markedly different biological behaviour and clinical outcomes [4]. These tumours demonstrate marked heterogeneity in biological behaviour, ranging from indolent low-grade lesions to aggressive adenocarcinomas with poor outcomes [5].

Mucinous neoplasms of the appendix are associated with significant morbidity when presenting with peritoneal dissemination. The most common clinical presentation in our cohort was acute appendicitis [6]. In our institution, more than 70% of patients with appendiceal neoplasms were initially referred from an outside hospital following appendectomy for acute appendicitis. Although several studies in the literature have evaluated the epidemiology, clinical presentation, pathological patterns, and prognostic factors of appendiceal neoplasms, data from the Indian subcontinent remain limited. Hence, we sought to study these patterns at a large-volume tertiary cancer centre in an Indian setting. The present study aims to analyse the clinicopathological characteristics of appendiceal neoplasms, evaluate overall survival as the primary treatment outcome, and compare outcomes within the low-grade appendiceal mucinous neoplasms (LAMN) subgroup (which constitutes the majority of cases).

## Materials and methods

Study design and setting

This was a single-centre, retrospective observational study conducted at a tertiary-level cancer centre that functions as a referral hospital for several states in India. A retrospective analysis was performed using data from the hospital database. The study was conducted in accordance with institutional ethical standards, and patient data were anonymised prior to analysis.

Sampling technique

Consecutive (total-enumeration) non-probability sampling was used. All consecutive patients with histologically confirmed appendiceal neoplasms managed between January 2021 and December 2025 who met the eligibility criteria were included.

Inclusion and exclusion criteria

Inclusion criteria were: (i) histologically confirmed appendiceal neoplasm; (ii) treatment at the study institution between January 2021 and December 2025; and (iii) availability of adequate clinical, pathological, treatment, and follow-up records. Exclusion criteria were: (i) inflammatory appendiceal conditions; (ii) negative appendectomy specimens; and (iii) incomplete clinical records or absent follow-up data.

Data collection and imaging

Clinical, pathological, treatment, and follow-up data were retrieved from medical records and institutional databases. Variables analysed included age, gender, histological subtype, disease stage, treatment modality, recurrence status, and survival outcomes. Imaging modalities used for diagnosis and staging included ultrasonography of the abdomen and pelvis, contrast-enhanced computed tomography (CECT), and/or magnetic resonance imaging (MRI) of the abdomen and pelvis. Perioperative mortality was defined as death occurring within 30 days of initial surgery. Staging has been adapted from the eighth edition of the American Joint Committee on Cancer (AJCC) staging system. Accordingly, primary tumors were classified as Tis, T1, T2, T3, T4a, or T4b based on the depth of invasion and involvement of adjacent structures, with LAMNs staged using the AJCC eighth Edition-specific criteria, including the Tis (LAMN) category for tumors confined to the appendiceal wall. Regional lymph node status was categorized as N0, N1a, N1b, N1c, N2a, or N2b, while distant metastasis was classified as M0, M1a (intraperitoneal acellular mucin), M1b (intraperitoneal metastasis containing tumor cells), or M1c (non-peritoneal distant metastasis). Overall stage grouping was assigned stages 0-IVC.

Classification and treatment

Patients were classified into subgroups based on histopathological type: LAMN, high-grade appendiceal mucinous neoplasm (HAMN), and adenocarcinoma [4]. Treatment was individualized according to the extent of disease and histopathological findings. Depending on these factors, patients underwent appendectomy, active surveillance following appendectomy, completion right hemicolectomy, or cytoreductive surgery (CRS) either with or without hyperthermic intraperitoneal chemotherapy (HIPEC) [7]. For patients undergoing HIPEC, mitomycin C was administered at a dose of 15 mg/m² at 43°C over 90 minutes for appendiceal mucinous neoplasms. For adenocarcinoma, cisplatin (50 mg/m²) combined with mitomycin C (15 mg/m²) was administered at 43°C over 60 minutes [8]. CRS included peritonectomy, omentectomy, bowel resection, colectomy, splenectomy, nephrectomy, and small-bowel resection with appropriate restoration of bowel continuity, as indicated [8]. Post-treatment follow-up included periodic clinical evaluation, serum tumour markers [carcinoembryonic antigen (CEA), CA-125, and CA 19-9], and CECT of the abdomen and pelvis as clinically indicated. Patients diagnosed with adenocarcinoma received adjuvant systemic chemotherapy with the capecitabine and oxaliplatin (CAPOX) regimen for six months.

Sample size

As this was a retrospective study of a rare malignancy, no a priori sample-size calculation was performed; the sample comprised all eligible patients presenting within the defined study period (N = 55). This convenience-based, all-comers approach is acknowledged as a limitation.

Statistical analysis

Descriptive statistics were used to summarise patient demographics and disease characteristics (median with 95% confidence interval for continuous variables; frequency and percentage for categorical variables). Overall survival was estimated using the Kaplan-Meier method, and survival distributions between groups were compared using the log-rank test. A Cox proportional-hazards model was used to estimate hazard ratios with 95% confidence intervals. A two-sided p-value < 0.05 was considered statistically significant. Analyses were performed using R version 4.x (R Foundation for Statistical Computing, Vienna, Austria) with the “survival” and “survminer” packages.

Ethical approval

The study was approved by the Institutional Review Committee (approval number IRC/2026/P-37) and conducted in accordance with the Declaration of Helsinki. Informed consent for treatment and open-access publication was obtained or waived by the committee.

## Results

Out of the 55 patients included in the study, the median age at presentation was 54 years (95% CI: 46-61), with a slight female predominance (30; 54.5%). The majority of patients had LAMN (37; 67.3%), followed by HAMN (8; 14.5%), adenocarcinoma (8; 14.5%), and signet-ring adenocarcinoma (2; 3.6%) (Table 1). Tumour stage was assigned in 41 patients; of these, 22 (53.7%) had stage IV disease at presentation.

**Table 1:** Patient and clinical characteristics CI: confidence Interval; CRS: cytoreductive surgery; LAMN: low-grade appendiceal mucinous neoplasm; HAMN: high-grade appendiceal mucinous neoplasm; HIPEC: hyperthermic intraperitoneal chemotherapy; TAH-BSO: total abdominal hysterectomy with bilateral salpingo-oophorectomy. Data are presented as n (%) unless otherwise specified.

Variables	Overall Cohort, N (%)
Age, median (95% CI)	54 (46–61)
Sex	
Female	30 (54.5)
Male	25 (45.5)
Histopathology	
LAMN	37 (67.3)
HAMN	8 (14.5)
Adenocarcinoma	8 (14.5)
Signet ring adenocarcinoma	2 (3.6)
Initial Presentation	
Appendectomy	24 (43.6)
Appendectomy + right hemicolectomy	1 (1.8)
Appendectomy + TAH-BSO	1 (1.8)
Ascitic fluid positive for malignancy	2 (3.6)
Intestinal obstruction	1 (1.8)
Pelvic mass	14 (25.4)
Peritoneal disease	2 (3.6)
Pseudomyxoma peritonei	3 (5.5)
Right hemicolectomy performed outside the institute	6 (10.9)
Tubo-ovarian mass	1 (1.8)
Tumor stage	N=41
Stage 0	8 (19.5)
Stage I	1 (2.4)
Stage II	7 (17.1)
Stage III	3 (7.3)
Stage IV	22 (53.7)
Surgery Performed After Initial Presentation	33 (60%)
Appendectomy	7(21.2)
Right hemicolectomy	20(60.6)
CRS	4(12.1)
CRS + HIPEC	2(6.1)

**Figure 1:**
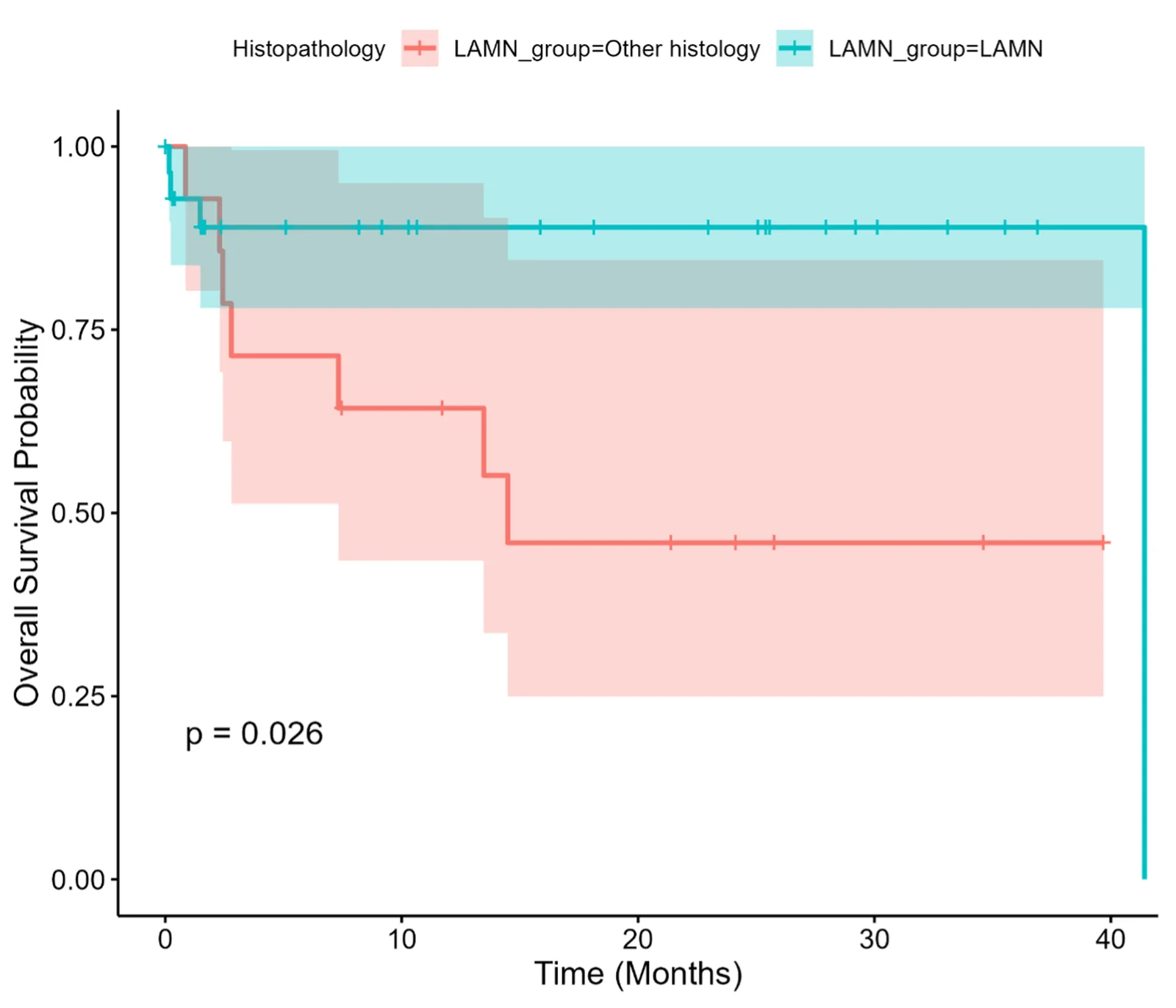
Kaplan-meyer curve for comparing overall survival between LAMN and other histology LAMN: low-grade appendiceal mucinous neoplasm.

Overall survival was evaluated between patients with LAMN and those with other histological subtypes using the log-rank test. The analysis demonstrated a statistically significant difference in survival (χ² = 4.98, p = 0.026). (Figure 1; Table 2). In the Cox proportional-hazards model, the coefficient (β) was −1.42, the hazard ratio (HR) was 0.24, and the p-value was 0.040, indicating that patients with LAMN had a hazard of death approximately 76% lower than that of patients with other histological types (reference group).

**Table 2:** One-, two-, and three-year overall survival comparison between LAMN and other histological types OS: overall survival; CI: confidence interval, LAMN: low-grade appendiceal mucinous neoplasm.

Group	Time(years)	OS(%)	Lower CI	Upper CI
Other histology	1	64.3	43.5	95
Other histology	2	45.9	25	84.5
other histology	3	45.9	25	84.5
LAMN	1	89	78	100
LAMN	2	89	78	100
LAMN	3	89	78	100

**Figure 2:**
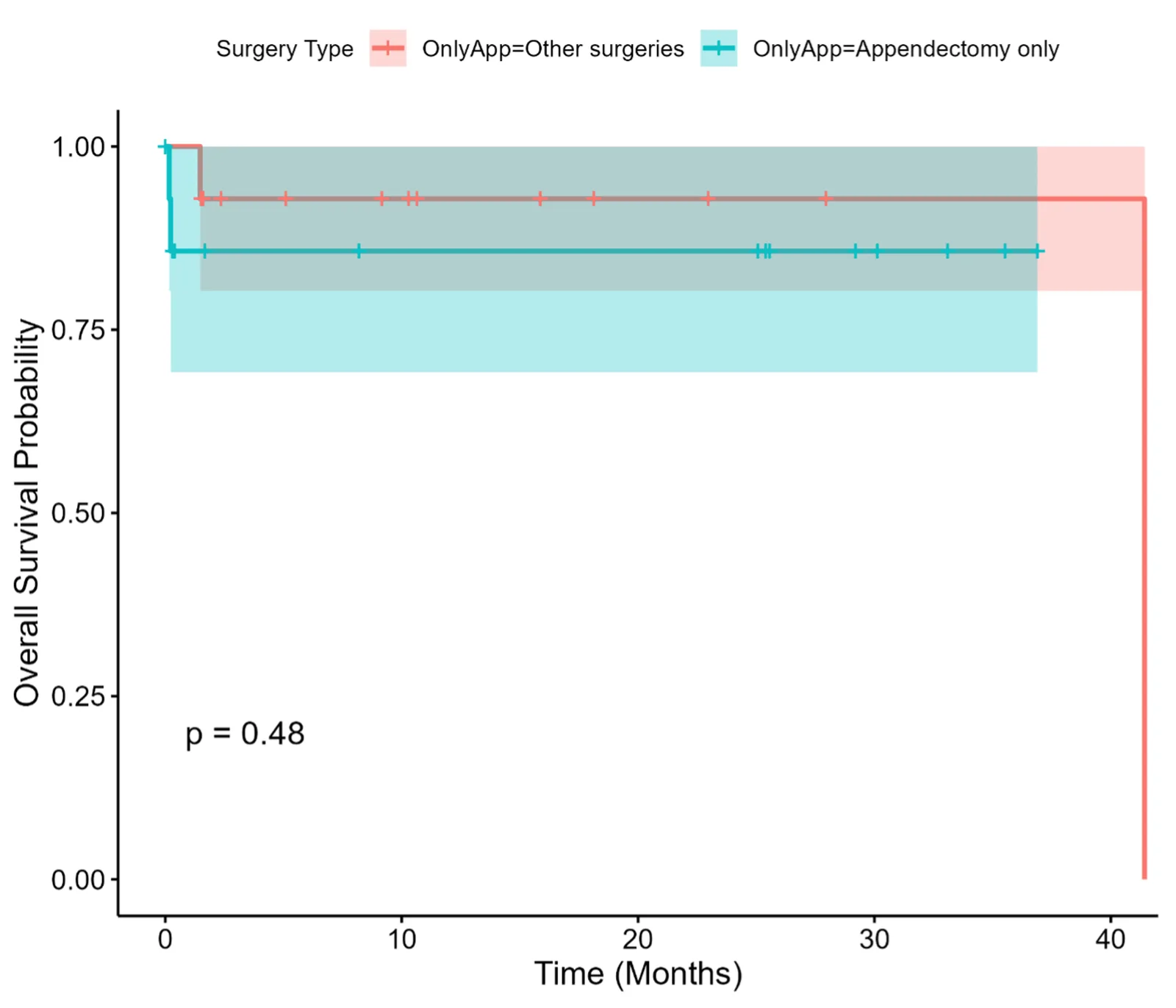
Kaplan.Meyer curve comparing overall survival between appendectomy vs other surgeries in LAMN group LAMN: low-grade mucinous neoplasm.

Among patients with LAMN, those who underwent appendectomy alone were compared with those undergoing other surgical procedures (right hemicolectomy, CRS, and CRS + HIPEC). Both groups had very few deaths (two events each). The mean survival time was slightly higher in the other-surgeries group (38.6 months) than in the appendectomy-alone group (35.5 months) (Table 3). One-, two-, and three-year overall survival was 92.9% in the other-surgeries group and 85.7% in the appendectomy-alone group; survival remained stable across the three years because very few deaths occurred. Comparison of overall survival between the two surgical groups using the log-rank test demonstrated no statistically significant difference (χ² = 0.49, p = 0.48) (Figure 2). Overall, among patients with LAMN, overall survival did not differ significantly between those undergoing appendectomy alone and those undergoing other surgical procedures; both groups demonstrated high survival rates (> 85%) up to three years.

**Table 3:** One-, two-, and three-year survival comparison between appendectomy and other surgeries in the LAMN group CI: confidence interval, LAMN: low-grade mucinous neoplasm.

Time (years)	Group	Survival (Probability)	Lower CI	Upper CI
1	Other surgeries	0.9285714	0.8030083	1
2	Other surgeries	0.9285714	0.8030083	1
3	Other surgeries	0.9285714	0.8030083	1
1	Appendectomy only	0.8571429	0.692117	1
2	Appendectomy only	0.8571429	0.692117	1
3	Appendectomy only	0.8571429	0.692117	1

## Discussion

Appendiceal neoplasms are rare but clinically significant tumours that comprise a heterogeneous group of epithelial and non-epithelial lesions. Across appendectomy series, these tumours are identified in roughly 0.2-2.5% of specimens [9]. They are often incidental findings, as their clinical presentation frequently mimics acute appendicitis, leading to diagnosis only after histopathological examination of the resected specimen. Population-based studies have also documented a rising incidence over recent decades, reflecting improved detection and reporting [2,3]. Several consensus systems have been developed to unify terminology and guide clinical practice [10].

The Peritoneal Surface Oncology Group International (PSOGI) and the European Reference Network for Rare Adult Solid Cancers (EURACAN) proposed a modern classification framework for appendiceal mucinous neoplasms [4,11]. This classification is now widely accepted and emphasises tumour grade and biological behaviour rather than purely anatomical criteria. Under this scheme, the epithelial group is the largest, comprising LAMN - the commonest lesion - together with HAMN and the mucinous, non-mucinous, and goblet-cell adenocarcinomas, whereas the non-epithelial group encompasses neuroendocrine tumours, neuroendocrine carcinoma, and mixed neuroendocrine-non-neuroendocrine neoplasms. Appendiceal tumours and pseudomyxoma peritonei (PMP) share a close association and a common clinical continuum [12]. PMP describes the intraperitoneal spread of an epithelial mucinous tumour, in which the peritoneal cavity fills with gelatinous ascites and mucinous implants; in most cases this follows perforation of a mucinous appendiceal tumour.

In the majority of cases, PMP arises from LAMN. PMP encompasses a spectrum of disease that ranges from benign mucinous ascites arising from low-grade appendiceal mucinous tumours to aggressive high-grade mucinous adenocarcinoma with extensive peritoneal involvement. Compared with other gastrointestinal malignancies, appendiceal neoplasms demonstrate distinct biological behaviour and metastatic patterns. The narrow lumen and thin wall of the appendix allow early perforation and access of its contents to the peritoneal spaces at an early stage of tumorigenesis. The epithelial cells of the appendix may be early or late in the adenoma-to-carcinoma transition, accounting for the wide variation in histological types of appendiceal epithelial tumours [13]. Moreover, the large colloid production of appendiceal mucinous neoplasms causes the redistribution phenomenon.

Survival outcomes after definitive treatment of a mucinous appendiceal neoplasm with peritoneal dissemination are highly variable and depend heavily on histological grade, the peritoneal cancer index (PCI), and the extent of completeness of cytoreduction, which is either CC-0 or CC-1. Several studies have demonstrated that completeness of CRS with HIPEC offers a prolonged survival benefit in appropriately selected patients [14]. Repeat cytoreductive surgery is considered a feasible option in selected patients with recurrent disease, provided adequate performance status and limited disease burden are present. Appendiceal peritoneal metastases display a further unique biological behaviour, often allowing prolonged survival even in the presence of recurrent peritoneal disease.

For epithelial tumours that are benign, an appendicectomy may be sufficient provided the surgical margin is free of disease [5].In contrast, patients with malignant appendiceal tumours have traditionally been managed with right hemicolectomy to facilitate regional lymph node clearance and address the possibility of occult nodal metastases. The sentinel lymph node concept proposed by Sugarbaker et al. has been used to guide the need for right hemicolectomy in patients with mucinous appendiceal tumours [8]. During the initial operation or at re-exploration, the appendiceal lymph nodes are carefully dissected from the posterior surface of the caecum. If metastatic involvement is demonstrated on intraoperative frozen-section examination, right hemicolectomy is subsequently performed.

Two subtypes of LAMN have been described by McDonald et al. [15]. In type 1, the process remains confined to the appendiceal lumen, whereas in type 2 mucin or neoplastic epithelium extends into the submucosa, wall, or periappendiceal tissue, with or without perforation; this second pattern carries the greater risk of peritoneal spread. For non-invasive mucinous tumours showing mucin or epithelial cells on the serosal surface but no peritoneal disease, a watch-and-wait strategy is advised, combining serial tumour markers with cross-sectional imaging every six to 12 months over five to 10 years [5].

Patients found, during surveillance following initial surgery for biopsy-confirmed appendiceal adenocarcinoma, to have residual appendiceal neoplasm should undergo a planned second-look operation, during which all peritoneal surfaces are carefully inspected. If peritoneal spread is detected, the patient should be treated with cytoreductive surgery, including excision of both the greater and lesser omentum, sampling of appendiceal lymph nodes (with right hemicolectomy performed if these nodes are positive), bilateral oophorectomy, and administration of HIPEC [7].

This single-centre retrospective study provides insight into the clinicopathological profile and outcomes of appendiceal neoplasms treated at a tertiary cancer centre in western India. Our findings demonstrate that overall survival in treated appendiceal neoplasms is predominantly influenced by tumour biology, particularly histological risk group and metastatic status at presentation. Patients with low-grade mucinous neoplasms experienced relatively favourable outcomes, often surviving without recurrence, whereas high-risk histological variants were associated with poorer survival and an increased incidence of peritoneal dissemination. Among patients with LAMN, overall survival did not differ significantly between those undergoing appendectomy alone for early-stage disease and those undergoing major surgical procedures for pseudomyxoma peritonei secondary to LAMN; both groups demonstrated high survival with very few deaths, reflecting the indolent tumour biology despite the stage of disease.

Our data are consistent with existing literature emphasising the prognostic importance of histology and disease extent in appendiceal malignancies [6,13]. Despite relatively favourable recurrence-free survival, overall survival remained modest, suggesting that advanced disease burden at presentation and the morbidity of major surgery contribute substantially to outcomes in this population. The lack of a statistically significant association with nodal status likely reflects limited nodal evaluation and small sample size rather than an absence of biological relevance.

Since this was a retrospective, single-institution, observational study with a relatively small sample size, it may limit the generalisability of the findings. Because of the study design and the limited number of events, multivariable analysis could not be performed. It is thus emphasised that larger, multicenter studies with adequate sample sizes to validate our findings and provide more robust evidence regarding the prognostic factors and outcomes of appendiceal neoplasms.

## Conclusions

Given the retrospective design and the limited number of events, which precluded multivariate analysis, these findings should be interpreted with caution. Nonetheless, the study offers a real-world representation of a rare malignancy managed at a high-volume tertiary cancer centre in India. Overall survival in treated appendiceal neoplasms was predominantly driven by tumour biology, with low-grade mucinous neoplasms showing favourable outcomes irrespective of the extent of surgery. Our findings support the need for a standardised protocol for the management of pseudomyxoma peritonei in the Indian population, informed by well-conducted meta-analyses and multi-institutional studies.
